# Prior cycles of anti-CD20 antibodies affect antibody responses after repeated SARS-CoV-2 mRNA vaccination

**DOI:** 10.1172/jci.insight.168102

**Published:** 2023-08-22

**Authors:** Hiromitsu Asashima, Dongjoo Kim, Kaicheng Wang, Nikhil Lele, Nicholas C. Buitrago-Pocasangre, Rachel Lutz, Isabella Cruz, Khadir Raddassi, William E. Ruff, Michael K. Racke, JoDell E. Wilson, Tara S. Givens, Alba Grifoni, Daniela Weiskopf, Alessandro Sette, Steven H. Kleinstein, Ruth R. Montgomery, Albert C. Shaw, Fangyong Li, Rong Fan, David A. Hafler, Mary M. Tomayko, Erin E. Longbrake

**Affiliations:** 1 Department of Neurology, and; 2 Department of Immunobiology, Yale School of Medicine, New Haven, Connecticut, USA.; 3 Department of Biomedical Engineering, Yale University, New Haven, Connecticut, USA.; 4 Yale Stem Cell Center and Yale Cancer Center, Yale School of Medicine, New Haven, Connecticut, USA.; 5 Yale Center for Analytical Sciences, Yale School of Public Health, New Haven, Connecticut, USA.; 6 Repertoire Immune Medicines, Cambridge, Massachusetts, USA.; 7 Quest Diagnostics, Secaucus, New Jersey, USA.; 8 Center for Infectious Disease and Vaccine Research, La Jolla Institute for Immunology, La Jolla, California, USA.; 9 Department of Medicine, Division of Infectious Diseases and Global Public Health, UCSD, La Jolla, California, USA.; 10 Department of Pathology, Yale School of Medicine, New Haven, Connecticut, USA.; 11 Program in Computational Biology and Bioinformatics, Yale University, New Haven, Connecticut, USA.; 12 Department of Internal Medicine,; 13 Section of Infectious Diseases, Department of Internal Medicine, and; 14 Department of Dermatology, Yale School of Medicine, New Haven, Connecticut, USA.

**Keywords:** Autoimmunity, COVID-19, Autoimmune diseases, Multiple sclerosis

## Abstract

**BACKGROUND:**

While B cell depletion is associated with attenuated antibody responses to SARS-CoV-2 mRNA vaccination, responses vary among individuals. Thus, elucidating the factors that affect immune responses after repeated vaccination is an important clinical need.

**METHODS:**

We evaluated the quality and magnitude of the T cell, B cell, antibody, and cytokine responses to a third dose of BNT162b2 or mRNA-1273 mRNA vaccine in patients with B cell depletion.

**RESULTS:**

In contrast with control individuals (*n* = 10), most patients on anti-CD20 therapy (*n* = 48) did not demonstrate an increase in spike-specific B cells or antibodies after a third dose of vaccine. A third vaccine elicited significantly increased frequencies of spike-specific non-naive T cells. A small subset of B cell–depleted individuals effectively produced spike-specific antibodies, and logistic regression models identified time since last anti-CD20 treatment and lower cumulative exposure to anti-CD20 mAbs as predictors of those having a serologic response. B cell–depleted patients who mounted an antibody response to 3 vaccine doses had persistent humoral immunity 6 months later.

**CONCLUSION:**

These results demonstrate that serial vaccination strategies can be effective for a subset of B cell–depleted patients.

**FUNDING:**

The NIH (R25 NS079193, P01 AI073748, U24 AI11867, R01 AI22220, UM 1HG009390, P01 AI039671, P50 CA121974, R01 CA227473, U01CA260507, 75N93019C00065, K24 AG042489), NIH HIPC Consortium (U19 AI089992), the National Multiple Sclerosis Society (CA 1061-A-18, RG-1802-30153), the Nancy Taylor Foundation for Chronic Diseases, Erase MS, the Robert Leet and Clara Guthrie Patterson Trust, and the Claude D. Pepper Older Americans Independence Center at Yale (P30 AG21342).

## Graphical abstract

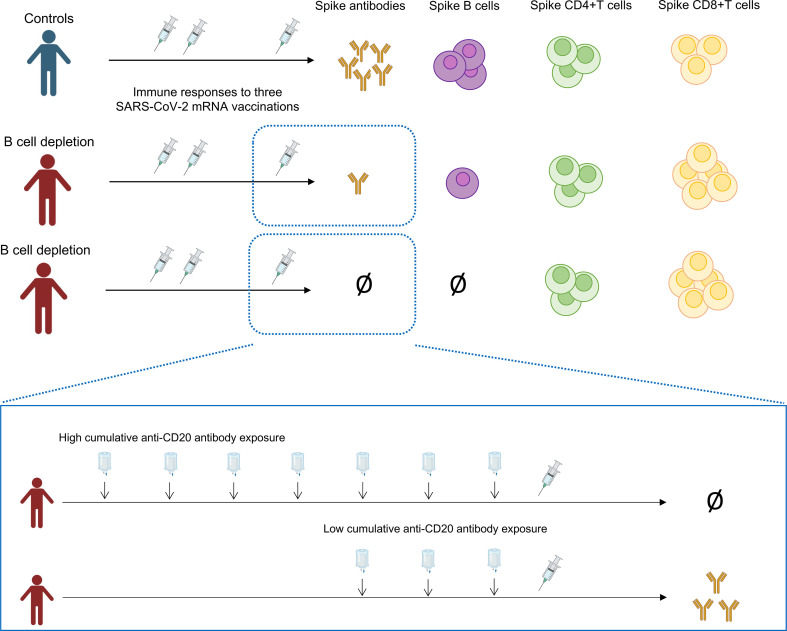

## Introduction

Severe acute respiratory syndrome coronavirus 2 (SARS-CoV-2) causes the clinical syndrome coronavirus disease 19 (COVID-19). Two mRNA vaccines targeting the full-length SARS-CoV-2 spike protein, BNT162b2 (Pfizer-BioNTech) and mRNA-1273 (Moderna), elicit humoral and cellular immunity and demonstrate high efficacy in preventing severe COVID-19 in healthy individuals (1–8). Waning vaccine effectiveness beginning around 6 months after vaccination in healthy individuals can be ameliorated by a booster vaccination (9).

In contrast with the immune response and protection afforded to healthy individuals, immunocompromised individuals have an attenuated response to vaccination (10–15). This is particularly true for B cell–depleted patients. Anti-CD20 mAbs, including rituximab and ocrelizumab, are utilized for a variety of autoimmune conditions and malignancies. These medications elicit loss of circulating immature and mature B cells, interrupting memory B cell generation and plasmablast/plasma cell differentiation. Most patients on B cell depletion therapy failed to mount an effective antibody response after 2 SARS-CoV-2 mRNA vaccinations (15–18), although they do develop virus-specific CD4^+^ and CD8^+^ T cells in numbers at or above the levels seen in healthy control individuals (15, 17).

Serial vaccination improved seroconversion rates for some subsets of immunocompromised patients (19), leading to a Center for Disease Control (CDC) recommendation that immunocompromised individuals receive an additional dose of vaccine for their primary series; however, it is not established whether this vaccination strategy is effective for B cell–depleted patients (20–22). Indeed, B cell–depleted patients have heterogeneous immune responses and there are insufficient data elucidating the vaccine response for a wide spectrum of these patients. Moreover, there is a need to identify the clinical characteristics of patients who are likely to benefit from serial vaccinations, with the goal of informing data-driven vaccination strategies for this vulnerable population.

Here, we evaluated immune responses to third mRNA vaccinations, measuring the antibody profiles against SARS-CoV-2 variants as well as the immunophenotype and functional profile for spike-specific T and B cells. We also applied systems biology approaches, including multiplex cytokine profiling, to clarify the relationship between proteomic signatures and vaccine immune responses (23–26).

## Results

### Attenuated humoral immune responses to third mRNA vaccinations in patients after anti-CD20 mAb.

**Figure 1:**
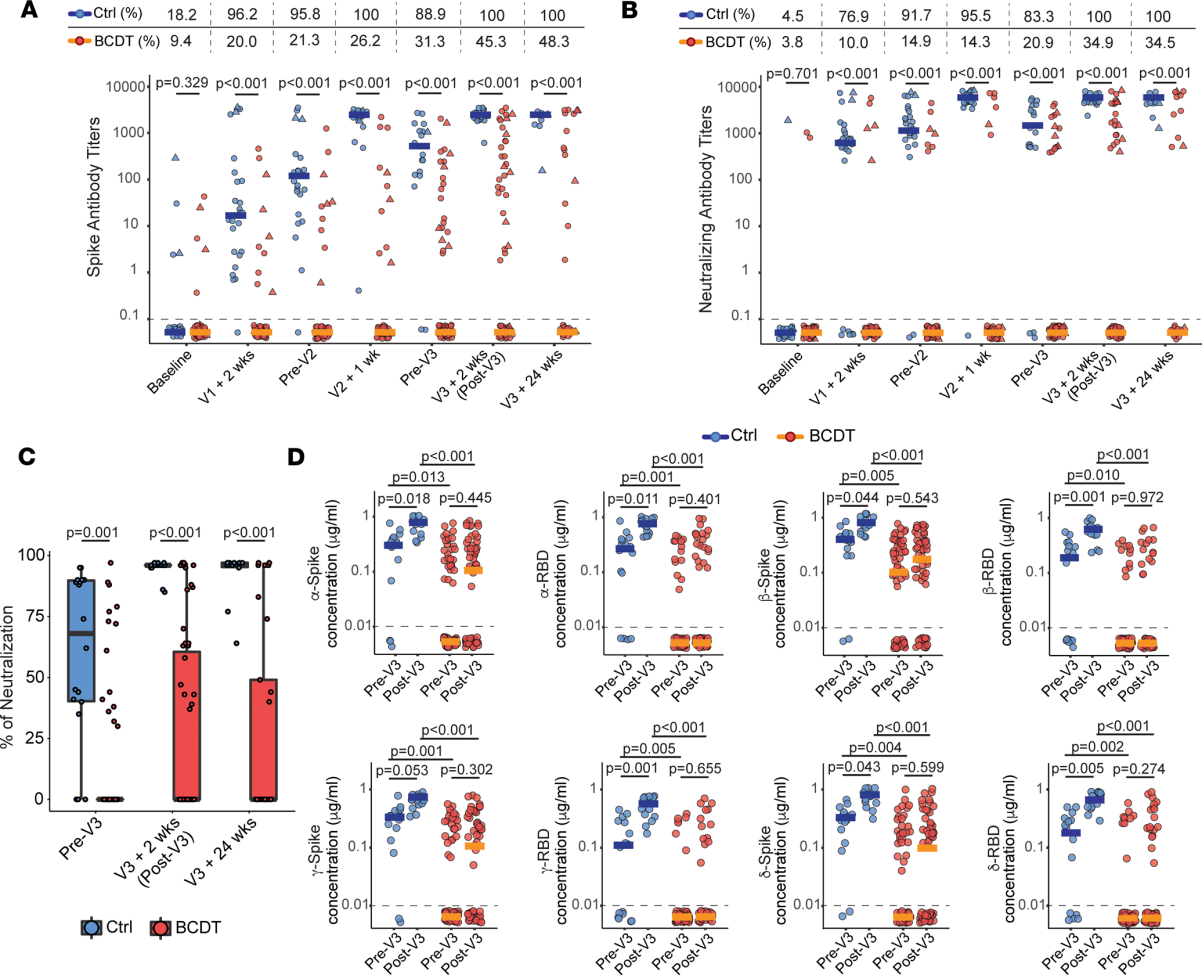
Humoral vaccine responses to third mRNA vaccines after anti-CD20 mAb treatments. (**A** and **B**) Dot plots of anti–SARS-CoV-2 spike antibody titers (**A**) and neutralizing antibody titers (**B**) were evaluated from before the first vaccine (baseline) to 6 months after the third vaccine (V3 + 24 weeks). The median is marked by a horizontal line. The proportion of seropositive participants at each time point is shown above the dot plots and the dotted line indicates the threshold for antibody detection (0.01 μg/mL). Circles represent participants without documented SARS-CoV-2 infections and triangles show those with known prior infection at each time point. Data were evaluated by independent-sample 2-tailed *t* test. (**C**) Box-and-whisker plots of the neutralization capacities between controls (pre-V3, *n* = 18; post-V3, *n* = 18; 24 weeks post-V3, *n* = 10) and B cell depletion (pre-V3, *n* = 67; post-V3, *n* = 63; 24 weeks post-V3, *n* = 29) is shown. The median is marked by a horizontal line, with whiskers extending to the farthest point within a maximum of 1.5 × IQR. Independent-sample 2-tailed *t* tests were performed. (**D**) Dot plots of anti-spike or anti-RBD antibody concentrations for each variant (Alpha, Beta, Gamma, and Delta) between controls (pre-V3, *n* = 16; post-V3, *n* = 15) and B cell–depleted participants (pre-V3, *n* = 66; post-V3, *n* = 59). The median is marked by a horizontal line. The proportion of seropositive participants at each time point is shown above the dot plots and the dotted line indicates the threshold for antibody detection (0.01 μg/mL). Independent-sample 2-tailed *t* tests with Bonferroni’s correction were performed. Ctrl, control participants; BCDT, patients with B cell depletion therapy.

To determine whether serial vaccination cumulatively increases humoral immunity and whether additional doses of vaccine boost protective antibodies in the context of B cell depletion, we conducted a prospective, longitudinal study that included B cell–depleted treated patients, disease-control individuals not on immunotherapy, and age- and sex-matched healthy individuals (Table 1). A total of 40 controls and 93 B cell–depleted patients contributed 492 samples at 7 different time points spanning 3 vaccinations. Sample sizes at each time point are presented in Supplemental Figure 1; supplemental material available online with this article; https://doi.org/10.1172/jci.insight.168102DS1 We observed an attenuated humoral immune response to mRNA vaccination in B cell–depleted patients across the initial 2-dose vaccine series; only 31.3% of these individuals had detectable spike antibodies before a third dose and seropositive individuals had significantly lower titers than controls (Figure 1A and Supplemental Figure 2A). The fraction of seropositive B cell–depleted individuals increased steadily over time; while only 26.2% of B cell–depleted patients were seropositive 1 week after 2 vaccines, 45.3% were seropositive after a third vaccine. The proportion of B cell–depleted patients producing neutralizing antibodies after 3 vaccines was more than double that observed after 2 vaccines (34.9% vs. 14.3%) (Figure 1, A–C, and Supplemental Figure 2, A and B). Receiving a third vaccination significantly increased spike antibody titers for controls (*P* < 0.001, pre-V3 vs. post-V3 in controls), and a similar trend was observed for seropositive B cell–depleted individuals (*P* = 0.055, pre-V3 vs. post-V3 in seropositive B cell–depleted individuals) (Supplemental Figure 2A). A few patients with B cell depletion therapies had a history of natural infection during serial vaccinations, which brought up the question of whether prior infection might be a predictor of vaccine serologic responses. Multivariable logistic regression analyses showed that after controlling for age, sex, BMI, and time from the last anti-CD20 mAb infusion, prior natural infection with SARS-CoV-2 was an independent predictor of seroconversion (Supplemental Figure 2, C and D). Intriguingly, lower cumulative exposure to anti-CD20 mAbs was also an independent predictor of producing spike and neutralizing antibodies across the B cell–depleted cohort.

Patients on B cell depletion therapy often fail to effectively clear SARS-CoV-2 after infection, which can lead to prolonged periods of viral replication, viral mutation, and new variant emergence within the same host (27–29). To determine the effect of vaccination on specific SARS-CoV-2 variants among healthy and B cell–depleted patients, we quantitated the antibody responses to specific viral strains (Figure 1D), employing a chip- and vacuum-based platform developed by our group (30). With this assay, we can evaluate the concentrations of up to 50 proteins simultaneously, including cytokines, chemokines, and specific antibodies. We confirmed that anti-spike antibodies measured using our multiplex platform correlate strongly with commercial immunoassays (Supplemental Figure 3). Vaccination elicited a significant increase in anti-spike and anti-RBD antibodies against Alpha (B.1.1.7), Beta (B.1.351), Gamma (P.1), and Delta (B.1.617.2) strains in control but not B cell–depleted individuals, demonstrating that the attenuation of vaccine-induced humoral immunity among these individuals was not strain specific (Figure 1D). We did not test for Omicron strains, as the majority of our sample collection preceded this variant’s emergence.

### Lower circulating B cell numbers limit the vaccine-induced rise in spike-specific B cells.

**Figure 2:**
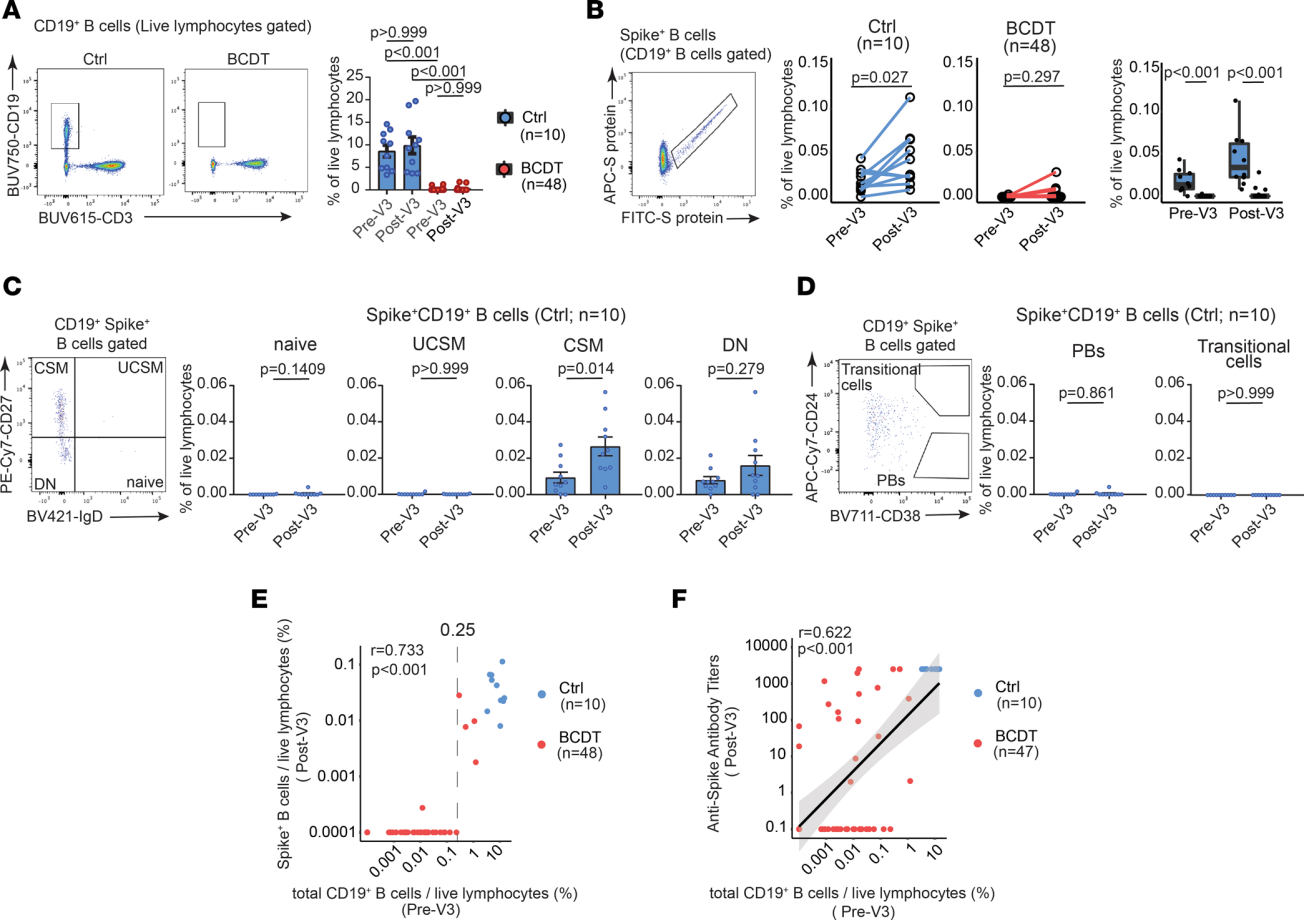
Spike-specific B cell response to third mRNA vaccines after B cell depletion. (**A**) Representative flow cytometry of CD19^+^ B cells and their proportions between controls (*n* = 10) and B cell–depleted participants (*n* = 48). Data are represented as mean ± SEM and independent-sample *t* tests with Bonferroni’s correction were performed. (**B**) Representative flow cytometry of spike^+^ B cells and their proportions between controls (*n* = 10) and B cell depleted (*n* = 48). Wilcoxon’s signed-rank test (middle) and independent-sample 2-tailed *t* tests with Bonferroni’s correction were performed. (**C** and **D**) Representative flow cytometry of each subset in spike^+^ B cells and their proportions between pre-V3 and post-V3 in controls (*n* = 10). UCSM, IgD^+^CD27^+^ non–class-switched memory B cells; CSM, IgD^–^CD27^+^ class-switched memory B cells; DN, IgD^–^CD27^–^ double-negative B cells. (**E** and **F**) Correlation between the proportion of CD19^+^ B cells before V3 and the proportion of spike^+^ B cells (**E**) or anti–SARS-CoV-2 spike antibody titers (**F**) after V3 in B cell–depleted participants (*n* = 47–48). The vertical dotted line represents the value of 0.25% (**E**). Linear regression is shown with 95% confidence intervals (gray area) and correlation statistics by 2-tailed Spearman’s rank correlation test were performed (**F**).

As expected, there were few total peripheral blood CD19^+^ B cells in B cell–depleted individuals (Figure 2A), and the presence of circulating B cells was positively correlated with longer intervals between most recent anti-CD20 infusion and vaccination, but not with the cumulative dose of anti-CD20 antibody treatment (Supplemental Figure 4, A and B). We quantified circulating spike-specific B cells before and after a third vaccine (pre- and post-V3); a third dose of mRNA vaccine elicited an increase in spike-specific B cells among control, but not B cell–depleted individuals (Figure 2B). These spike-specific B cells were primarily IgD^–^CD27^+^ class-switched memory B cells and IgD^–^CD27^–^ double-negative (DN) cells (31) (Figure 2, C and D, and Supplemental Figure 5A). Among the DN cells, most were CD11c^–^CXCR5^+^ DN1 memory precursor cells, but CD11c^+^CXCR5^–^ DN2 activated extrafollicular naive B cells were detected as well (Supplemental Figure 5B). The presence of CD19^+^ B cells in the peripheral blood before V3 was strongly predictive of the generation of spike-specific B cells after V3 (*r* = 0.733, *P* < 0.001), and all individuals whose B cells comprised more than 0.25% of peripheral lymphocytes generated spike-specific B cells after a third vaccine (Figure 2E). Intriguingly, while a positive correlation between total peripheral blood B cells and spike-antibody titers was observed (*r* = 0.622, *P* < 0.001), some B cell–depleted patients produced anti-spike antibodies despite having few peripheral blood B cells before V3 (Figure 2F). Comparing the clinical information between antibody producers (seropositive individuals after V3) and antibody nonproducers (seronegative after V3), the former had significantly lower cumulative exposure to anti-CD20 mAbs (Table 2). In contrast, there was no significant difference between the duration from the last anti-CD20 antibodies to a third vaccine. We also evaluated the relationship between circulating immune cell subtypes and neutralizing antibodies (Supplemental Figure 6). While total circulating B cells and neutralizing-antibody titers were positively correlated (*r* = 0.5930, *P* < 0.001), we again observed that some B cell–depleted patients produced neutralizing antibodies despite low numbers of circulating B cells. B cell–depleted individuals with measurable neutralizing antibodies had a lower cumulative exposure to anti-CD20 mAbs, similar to those with measurable total anti-spike antibodies. Overall, detection of CD19^+^ B cells in the blood may be a useful biomarker for predicting the induction of spike-specific B cells.

### Robust increase in spike-specific T cells after third vaccinations.

**Figure 3:**
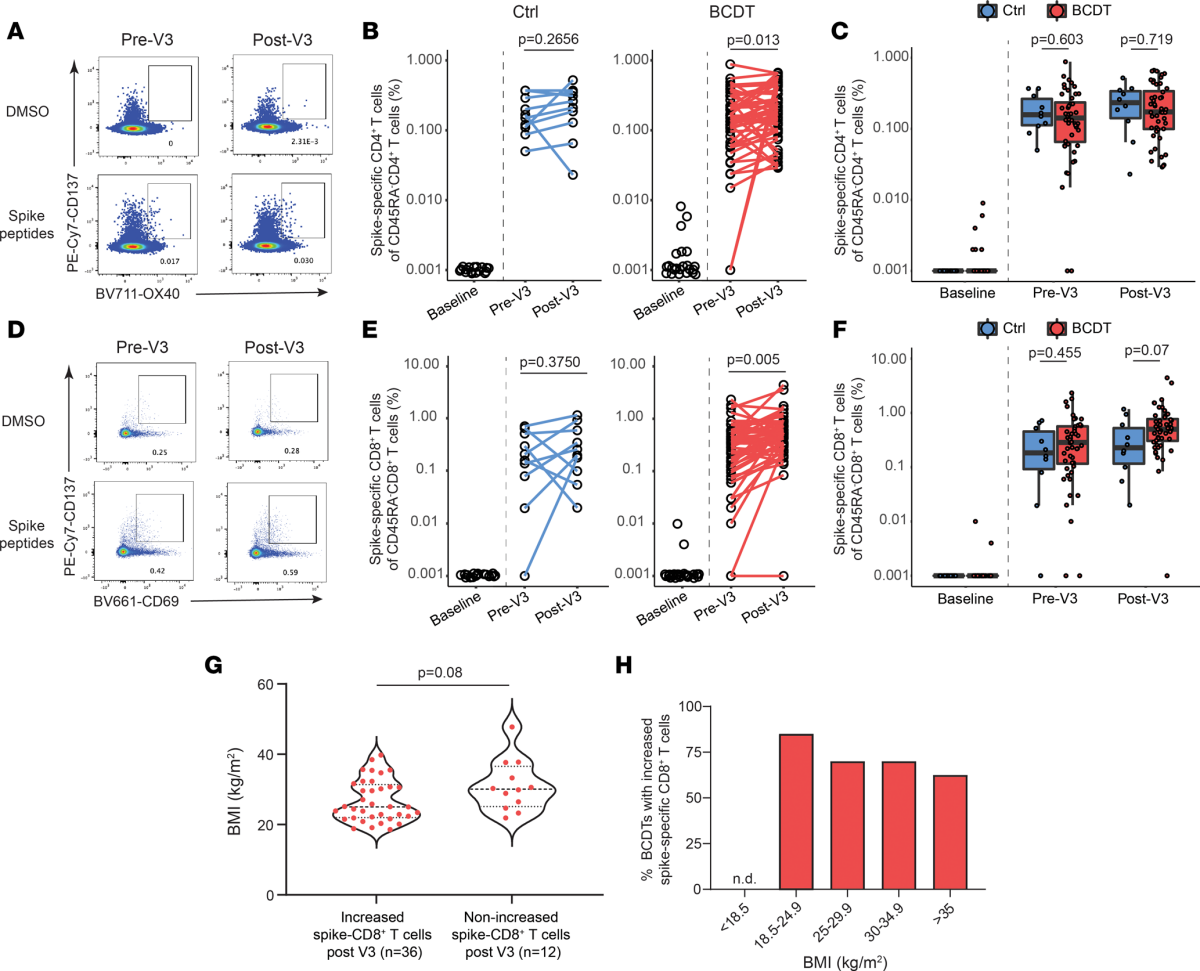
Spike-specific T cell response to third mRNA vaccine after anti-CD20 mAb therapy. (**A**–**C**) Representative flow cytometry of CD137^+^OX40^+^ spike-specific CD4^+^ T cells (**A**) and their proportions between controls (*n* = 10) and B cell–depleted participants (*n* = 48) in relation to a third vaccination (**B** and **C**). Baseline, prevaccination samples for controls (*n* = 17), and B cell–depleted participants (*n* = 23) were also evaluated. Wilcoxon’s signed-rank test (**B**) and 2-tailed independent-sample *t* test (**C**) were performed. (**D**–**F**) Representative flow cytometry of CD137^+^CD69^+^ spike-specific CD8^+^ T cells (**D**) and their proportions between controls (*n* = 10) and B cell depletion (*n* = 48) (**E** and **F**). Wilcoxon’s signed-rank test (**E**) and 2-tailed independent-sample *t* test (**F**) were performed. (**G**) Body mass index (BMI, kg/m^2^) between B cell–depleted participants with increased spike-specific CD8^+^ T cells (*n* = 36) and without an increase (*n* = 12). Two-tailed independent-sample *t* test was performed. (**H**) The proportion of increased spike-specific CD8^+^ T cells before V3 based on BMI (kg/m^2^). WHO BMI classification was applied for the subgroups with B cell depletion: BMI < 18.5 (*n* = 0), 18.5–24.9 (*n* = 20), 25.0–29.9 (*n* = 10), 30.0–34.9 (*n* = 10), > 35.0 (*n* = 8).

As robust T cell responses have been reported in B cell–depleted patients after 2 mRNA vaccinations (15), we monitored both spike-specific CD4^+^ memory T cells and CD8^+^ memory T cells before and after a third vaccine. Both groups exhibited robust spike-specific T cells months after their second vaccine (pre-V3). Although the duration between the second and third vaccines was significantly shorter for B cell–depleted individuals compared with controls (Supplemental Table 1), this interval was not correlated with the proportions of spike-specific T cells prior to the third vaccine in our cohort (Supplemental Figure 7). The proportion of these T cells significantly increased after V3 in B cell–depleted patients, but not in controls (Figure 3, A–F). The overall proportions of spike-specific CD4^+^ and CD8^+^ T cells was not significantly different between controls and B cell–depleted individuals, although there were trends for a heightened CD8^+^ response in these patients only (Figure 3F). To elucidate which clinical variables were associated with vaccine-associated increases in spike-specific memory CD8^+^ T cells for B cell–depleted treated individuals, we applied logistic regression models (Table 3) and found that lower body mass index (BMI, kg/m^2^) correlated with increased spike-specific memory CD8^+^ T cells (Figure 3, G and H). Natural infection with SARS-CoV-2 prior to a third vaccination was not correlated with the change in spike-specific CD8^+^ T cells elicited by a third vaccination (*P* = 0.56, Fischer’s exact test, data not shown).

### Serum proteomics after third vaccine in B cell–depleted patients.

Systems biology approaches have been effectively used to explore the molecular determinants of vaccination responses and some proteomic signatures are reported to be linked to various vaccine components (23–26). Immune cell proteomics, including cytokine/chemokine expression, have not yet been evaluated after vaccination in individuals using B cell depletion. To determine whether serum proteomics could detect differences in the immune responses of control and B cell–depleted individuals, we used our multiplex platform described above (30). There were no observed changes in systemic inflammatory cytokines/chemokines after vaccination for either control or treated patients (Supplemental Figure 8), and there were no clear differences between B cell–depleted patients who did or did not mount detectable humoral immune responses (Supplemental Figure 9). Among B cell–depleted individuals with mounted spike-specific CD4^+^ T cell expansion after the third vaccination, we observed a significant decrease in von Willebrand factor (vWF) after V3 (Supplemental Figure 10). Moreover, CCL5 was significantly decreased in B cell–depleted individuals who manifested increased spike-specific T cell responses (Supplemental Figures 10 and 11). These results indicate that proteomic signatures were related to T cell responses in individuals after anti-CD20 mAb therapy.

### Prior cycles of anti-CD20 antibodies affected humoral immunity to serial COVID-19 vaccination.

**Figure 4:**
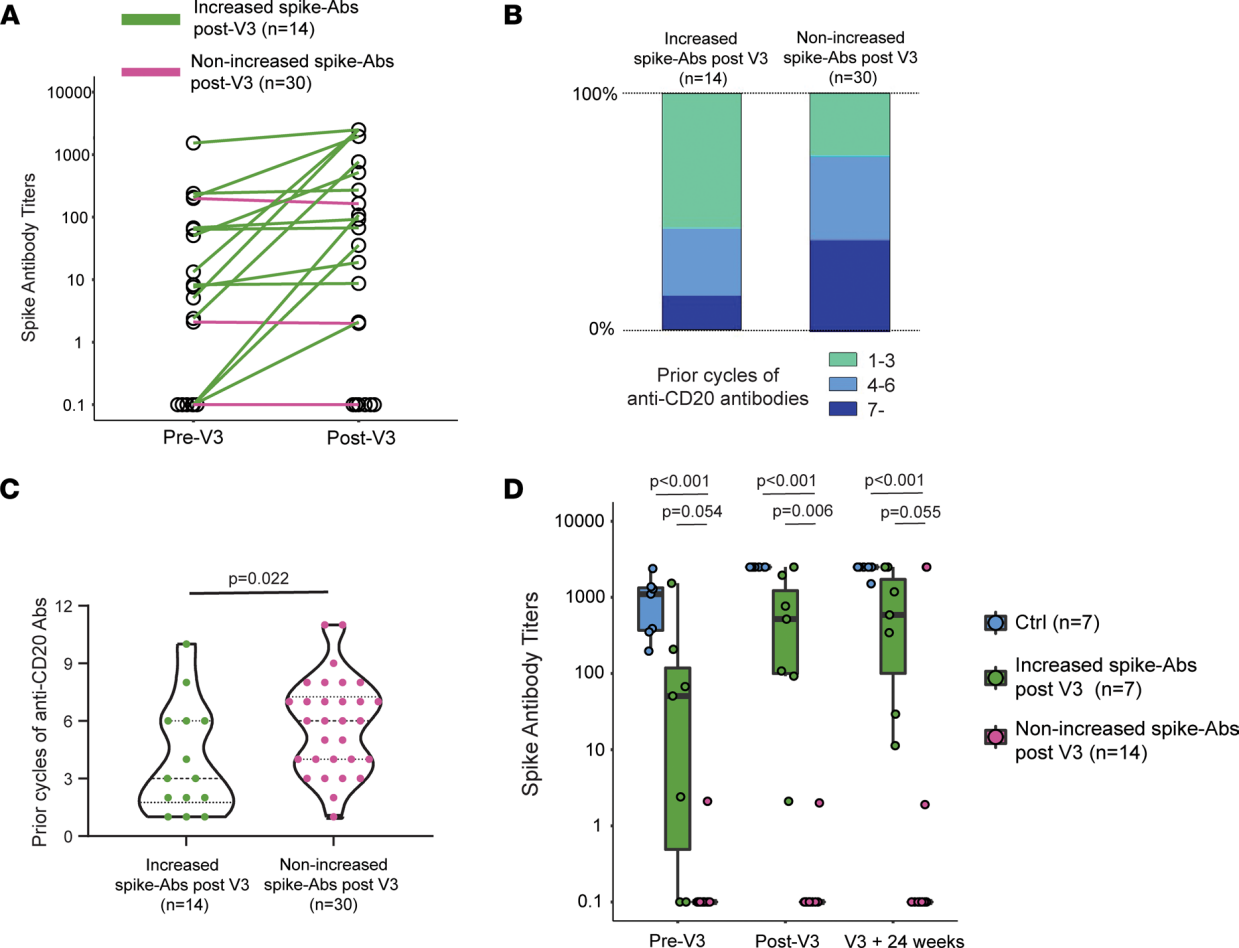
The prediction of humoral immune responses after third vaccine in B cell–depleted participants. (**A**) Sequential anti–SARS-CoV-2 spike antibody titers before and after V3 in B cell–depleted participants (*n* = 44). (**B** and **C**) Prior cycles of anti-CD20 antibodies between B cell–depleted participants with increased anti-spike antibodies (*n* = 14) and without an increase (*n* = 30). Two-tailed independent-sample *t* test was performed (**C**). (**D**) Box-and-whisker plots of sequential anti–SARS-CoV-2 spike antibody titers from before V3 to 24 weeks after the third vaccine (V3 + 24 weeks). The median is marked by a horizontal line, with whiskers extending to the farthest point within a maximum of 1.5 × IQR. Controls (blue, *n* = 7), B cell–depleted participants who increased anti-spike antibodies after V3 (green, *n* = 7), and B cell–depleted participants who did not increase anti-spike antibodies after V3 (purple, *n* = 14) are shown. Independent-sample 2-tailed *t* tests with Bonferroni’s correction were performed.

We used logistic regression to elucidate which B cell–depleted individuals were likely to mount a humoral immune response to a third anti-SARS-CoV-2 vaccination. We observed increasing anti-spike antibody titers after V3 for 14 of 44 patients; of the remaining patients, 2 had slightly decreasing titers (2.1 U/mL to 2 U/mL, and 199 U/mL to 164 U/mL) while the rest never mounted detectable anti-spike antibodies (Figure 4A). Multivariable logistic regression demonstrated that longer time since last anti-CD20 treatment and a lower cumulative dose of anti-CD20 antibodies (lower number of prior treatment cycles) were independently associated with greater odds of serologic response (i.e., being “responder”) to a third vaccine (Table 4, Figure 4, B and C, and Supplemental Figure 12). There was no significant association between known SARS-CoV-2 infections prior to a third vaccine and increased spike-antibodies after vaccination (*P* = 0.58, Fischer’s exact test). Finally, we evaluated the durability of a detectable serologic response to third-vaccine immunity over time. Six months after V3, both controls and seropositive B cell–depleted patients maintained a robust humoral response to SARS-CoV-2 (Figure 4D). These data suggest that serologic “responders” could maintain humoral responses for at least 6 months after V3.

## Discussion

Although serial vaccination improves seroconversion rates for some immunocompromised patients, the effectiveness of this strategy has not yet been established for B cell–depleted patients. Despite this, most of these patients have received at least 4 vaccinations. Many are reporting vaccine burn-out, frustration with ever-changing recommendations, and reluctance to get yet another vaccine. Others continue to fear infection and seek out any opportunity for enhanced protection against COVID-19, sometimes getting more vaccines than recommended. Evidence-based decision-making tools are needed. Our study represents the first comprehensive assessment to our knowledge of the serologic and cellular immune responses, combined with systemic proteomics, elicited by a third mRNA vaccine.

Multivariate regression models identified longer intervals between anti-CD20 infusions and vaccination and lower cumulative exposure to anti-CD20 antibodies as being independently associated with increasing anti-spike antibody titers after V3 in B cell–depleted patients. Longer intervals between anti-CD20 mAb and vaccination have been previously correlated with improved humoral responses (12, 32, 33). Previously published data have also shown that peripheral B cell counts predict vaccine-induced seroconversion among B cell–depleted patients (34–36); in our cohort, we found a stronger association between peripheral B cells and spike-specific B cells (Figure 2E) than between total B cells and spike antibody titers (Figure 2F). This difference may be due in part to unique aspects of the study design, including the time points studied and the cohort demographics.

We were able to identify cumulative exposure to anti-CD20 medication, along with the time between B cell depletion and vaccination, as important predictors of those for whom serial vaccination elicits a humoral response (Figure 2F). This suggests that an impaired vaccine response may be a specific risk for those on long-term B cell depletion. B cell–depleting strategies are utilized chronically for treating a variety of autoimmune disorders, including multiple sclerosis (MS), rheumatoid arthritis, and autoimmune blistering disease (37, 38). While these medications generally have a favorable side effect profile (39), long term use has also been associated with prolonged delays in B cell reconstitution (40) and an increased risk of hypogammaglobulinemia (41). Although anti-CD20 therapy rapidly depletes circulating B cells, B cells within secondary lymphoid tissue can be resistant to depletion (42–44). Thus, the decreased vaccination response observed in patients on long-term therapy may represent complex alterations in immune function capabilities. Further investigation will be needed to elucidate the relationship between repeated B cell depletion, tissue-resident B cells, and vaccine responses.

We also evaluated cellular responses to mRNA vaccines and demonstrated a robust spike-specific CD4^+^ and CD8^+^ T cell response for both B cell–depleted and control patients. Although the T cell response was largely sustained between the initial vaccine series and the third dose, the spike-specific CD8^+^ T cell response was enhanced in B cell–depleted patients compared with controls (Figure 3C), corroborating previous reports (45, 46). These data together provide evidence in human systems that cell-cell interactions are regulated thoroughly and imply a possible role for B cells in the regulation of CD8^+^ T cell expansion. Further experiments to examine the mechanisms for this observation are warranted. Multivariate analysis using backward binary logistic regression identified higher BMI (kg/m^2^) as being significantly associated with attenuated CD8^+^ T cell responses to serial vaccination (Figure 3G). Obesity increases the likelihood of a poor vaccine-induced immune response (47, 48) and both human data and mouse models have shown not only poorer seroconversion rates, but also impaired T cell responses to influenza vaccinations in obese individuals (49–52). Putative mechanisms include obesity-induced alterations in CD8^+^ T cell–mediated metabolism and effector functions (53); additionally, antigen-presenting capacities of dendritic cells are impaired in obesity, with downstream impairments in CD8^+^ T cell activation (54). Our findings indicate obesity as a potential risk factor for diminished vaccine responses among B cell–depleted patients.

To better understand how vaccination affects the systemic immune response, we evaluated proteomics before and after a third vaccine using high-plex immune assays. While we did not observe differences in circulating protein expression between controls and anti-CD20 mAb–treated patients, a lower vWF concentration was detected in B cell–depleted patients who increased CD4^+^ T cell responses after a third vaccine. Moreover, a significant decrease in CCL5 was detected in individuals with higher T cell responses (Supplemental Figures 10 and 11). These factors are released from several cell subsets, including macrophages, endothelial cells, platelets, and fibroblasts (55, 56). The mRNA vaccines are reported to induce platelet activation (57), and furthermore enhance innate and CD8^+^ T cell responses through type I interferon–dependent MDA5 signaling (58). Although further investigation is needed to elucidate the exact mechanisms affecting vaccine responses, these results demonstrate that immunomodulatory capacities of mRNA vaccines differ among B cell–depleted individuals and that comprehensive proteomics analysis might be useful to predict better immune responses to mRNA vaccinations.

There are some limitations to our study. Our data disproportionately represent individuals of European ancestry and those with primary autoimmune diseases, mainly MS, which may affect our results. Although data from control individuals showed a robust cellular and humoral response to vaccination, aligning well with previously published cohorts (59, 60), in our study, controls were fewer in number than B cell–depleted participants and this could be a limitation. Moreover, this study partially overlapped with the Omicron (B.1.1.529) surge, and some participants may have been asymptomatically infected during the study. Interestingly, one B cell–depleted patient who failed to develop anti-spike Abs in response to vaccination did seroconvert after a natural SARS-CoV-2 infection 5 months after their third vaccination. This case shows that a poor response to 3 doses of mRNA vaccine does not mean failure of the humoral immune response to SARS-CoV-2. Another important consideration is that most B cell–depleted patients who received mRNA-1273 vaccinations for a third dose received full doses (rather than the half-doses typically administered as “boosters” to immunocompetent individuals). The immune consequences of B cell depletion in patients are complex and may be impacted by many variables, including timing of anti-CD20 dosing, concentration of the stimulus, and type of immune stimulation such as vaccination versus infection. Nevertheless, despite repeated dosing, even high-dose mRNA vaccines elicited a humoral anti-spike antibody response in only a minority of B cell–depleted patients.

In summary, our data implicate cumulative anti-CD20 antibody dose, time since last anti-CD20 antibody infusion, and BMI as variables useful for the prediction of immune responses in patients with B cell depletion. These data provide a potential framework for assessing the likelihood of a future immune response to repeated vaccinations among B cell–depleted patients and shaping policy-level recommendations to this vulnerable population.

## Methods

### Patients and samples.

We recruited adult patients (≥18 years) who received vaccination against COVID-19 between February 2021 and May 2022. Study participants had a diagnosis of autoimmune neurologic or skin disease, either treated with B cell depletion, or on no immunotherapy. Patients without any therapies (disease controls) and healthy individuals were classified as controls and participants on B cell depletion therapies were classified into the B cell depletion therapy group. Those who were pregnant, had received high doses of steroids within 1 month of vaccination, or had newly initiated anti-CD20 medications (first dose within 2 weeks of vaccination) were excluded. Participants who donated baseline samples but not postvaccination samples were removed from the analyses. Participants donated blood prior to vaccination, and then at prespecified time points thereafter (Supplemental Figure 1). Additional participants were recruited at the time of third vaccinations, such that not all participants studied before and after a third vaccine had provided a baseline sample prior to any COVID-19 vaccination. Demographics for the subgroup studied before and after V3 are reported in Supplemental Table 1.

### Blood processing.

Peripheral blood mononuclear cells (PBMCs) were prepared from whole blood by Ficoll gradient centrifugation with Lymphoprep (Stemcell), counted (TC20 Automated Cell Counter, Bio-rad), resuspended in serum-free Bambanker medium (Bulldog-bio) at 10 million cells/mL, and stored in liquid nitrogen. Serum was isolated, aliquoted, and stored at –80°C.

### The measurements of anti–SARS-CoV-2 antibodies.

Anti-spike and neutralizing SARS-CoV-2 antibodies were measured by Quest Diagnostics (test code 39820) and SARS-CoV-2 Surrogate Virus Neutralization Test Kit (GenScript). Strain-specific antibodies were measured using our high-plex immune-serology assay (below).

### Flow cytometry.

PBMCs were resuspended in Live/Dead Fixable Blue Dead Cell Stain (Thermo Fisher Scientific), blocked with Human TruStan FcX (BioLegend), and stained. Lymphocyte events were acquired by BD FACSymphony A5 and data analysis was done with BD FACSDiva Software. The antibodies were as follows: anti-CD3 (BD Biosciences, 751252, SK7), anti-CXCR5 (BD Biosciences, 558113/565191, RF8B2), anti–HLA-DR (BD Biosciences, 564040, G46-6), anti-CD4 (BD Biosciences, 612936, SK3), anti-CD69 (BD Biosciences, 750213, FN50), anti-CCR7 (BD Biosciences, 566437, 3D12), anti-CD45RA (BD Biosciences, 560674, HI100), anti-IgD (BioLegend, 348226, I-A6), anti-CD11c (BioLegend, 301636, 3.9), anti-CD38 (BioLegend, 303528, HIT2), anti-CD19 (BioLegend, 302262, HIB19), anti-CD21 (BioLegend, 354904, Bu32), anti-CXCR3 (BioLegend, 353736, G025H7), anti-CD27 (BioLegend, 356412, M-T271), anti-CD24 (BioLegend, 311132, ML5), anti–IFN-γ (BioLegend, 502532, 4S.B3), anti-CD25 (BioLegend 302632, BC96), anti-OX40 (BioLegend, 350030, Ber-ACT35), anti-CD8 (BioLegend, 44756, SK1), anti-CD38 (BioLegend, 356610, HB-7), anti–PD-1 (BioLegend, 329906, EH12.H7), anti-CD40LG (BioLegend, 310840, 24-31), anti-CD137 (BioLegend, 309818, 4B4-1), and recombinant SARS-CoV-2 spike protein (R&D Systems, AFR10561-020/AFG10561-020).

### The detection of SARS-CoV-2–specific T cells.

PBMCs were diluted with RPMI to 0.5 × 10^6^ cells/mL and rested overnight (16–18 hours) at 37°C in a 5% CO_2_ incubator. Cells were incubated with anti-CD40 blocking antibodies (catalog 130-094-133, HB14, Miltenyi Biotec; 0.5 μg/mL) and then stimulated with a spike pool composed of 15-mer peptides overlapping by 10 amino acids resuspended in DMSO (final concentration, 1 μg/mL) (55) and anti–human CD28 and CD49d antibodies (catalog 347690, BD Biosciences; 1 μg/mL) for 24 hours. As a negative control, an equimolar amount of DMSO was added. For the last 8 hours, protein secretion inhibitors (catalog 420601/420701, BioLegend) were added. Cells were washed, incubated with Zombie Aqua Fixable Viable kit (catalog 423102, BioLegend) for viability, and then blocked with Human TruStan FcX (catalog 422302, BioLegend). Surface-staining antibodies were directly added and incubated for 45 minutes at 37°C. Cells were then fixed, permeabilized with permeabilization buffer (catalog 554723, BD Biosciences), and stained with intracellular staining antibody cocktails for 45 minutes at 4°C. Cells were washed with permeabilization buffer (Invitrogen) and events were acquired by BD FACSymphony A5. Antibodies utilized are listed under *Flow cytometry*. Activated CD4^+^ T cells were defined by dual expression of CD137 and OX40, and activated CD8^+^ T cells were identified by dual expression of CD137 and CD69. All data from these assays were background subtracted using paired DMSO control samples. Spike peptides were provided from the La Jolla Institute for Immunology. The demographic data for the patients who donated samples for these assays are shown in Supplemental Figure 1 and Supplemental Table 1.

### Workflow of high-plex immune-serology assay.

We adopted a newly developed, high-plex immune-serology assay to measure circulating proteins and strain-specific anti–SARS-CoV-2 IgG binding antibodies (30). In brief, a pair of polydimethylsiloxane (PDMS) microfluidic devices were introduced on the same poly-L-lysine–coated slide (PLL slide) prepared by conventional soft lithography process. The first PDMS device had 5-turn serpentine patterns, and perpendicularly aligned microfluidic channels in the second PDMS device can allow obtaining 5 replicates in a single assay. Capture antibodies and each recombinant SARS-CoV-2 antigen were introduced in each inlet. For the signal detections, a mixture of biotinylated detection antibodies and PE-conjugated anti–human IgG antibody (catalog ab7006, Abcam) was loaded onto the barcoded array chip for 45 minutes at room temperature. Afterward, APC-conjugated streptavidin (catalog 17-4317-82, Thermo Fisher Scientific) and BSA solution were applied at room temperature sequentially. Fluorescence images were obtained using a Genepix 4200A scanner (Molecular Devices). The mean photon counts were evaluated from intersection of columns and rows by aligning a 20 × 20 μm^2^ square array template in Genepix Pro 6.1 software (Molecular Devices). Only values higher than the threshold from more than 3 out of 5 replicates were collected by log_2_ normalization after subtracting the background threshold. Afterward, intensity values were converted to concentration using the titration curve.

### Statistics.

Descriptive statistics were summarized as frequencies and percentage, means and SD, or medians and IQR if not normally distributed. For categorical variables, differences between patients receiving B cell depletion therapy and controls were evaluated using χ^2^ test or Fisher’s exact test as appropriate. Independent-sample 2-tailed *t* test was used to compare the continuous variables by group. When comparing the post-V3 and pre-V3 changes within the same participant, Wilcoxon’s signed-rank test was used. Spearman’s correlation analysis was performed to examine the relationship between the humoral immune responses (anti-spike^+^ B cells or anti-spike antibody titers) and the percentage of CD19^+^ B cells among patients and controls. Multivariate logistic regression models were built to predict the likelihood of having anti-spike antibody increase or spike-specific CD8^+^ T cells increase after V3 among patients with B cell depletion therapy. Due to the small sample size, 5 predictors were chosen based on descriptive analyses and literature: age, sex, BMI, time from last anti-CD20 antibody infusion to V3 immunization, and number of prior cycles of anti-CD20 antibody infusion. All statistical analyses were performed using R, SAS 9.4, or Prism 9 (GraphPad Software). A *P* value of less than 0.05 was considered significant unless otherwise stated. Detailed information about statistical analysis, including tests and values used, is provided in the figure legends.

### Study approval.

This study was approved by the Institutional Review Board at the Yale School of Medicine. Written informed consent was obtained from all enrolled patients and healthy donors.

### Data availability.

The data reported in this paper are provided in Supporting Data Values. Additional data are available from the corresponding authors upon request.

## Author contributions

The experiments were conceptualized by HA, DAH, MMT, and EEL. HA, DK, RL, MKR, JEW, and TSG performed the experiments and analyzed data. NL, NCBP, IC, KR, and WER organized the samples and clinical information. AG, DW, and AS prepared for peptides for experiments. KW and FL did the statistical analyses. HA, MMT, and EEL wrote the original draft. SHK, RRM, ACS, RF, DAH, MMT, and EEL supervised the experiments. All the authors reviewed the manuscript.

## Supplementary Material

Supplemental data

ICMJE disclosure forms

Supplemental table 1

Supporting data values

## Figures and Tables

**Table 1:**
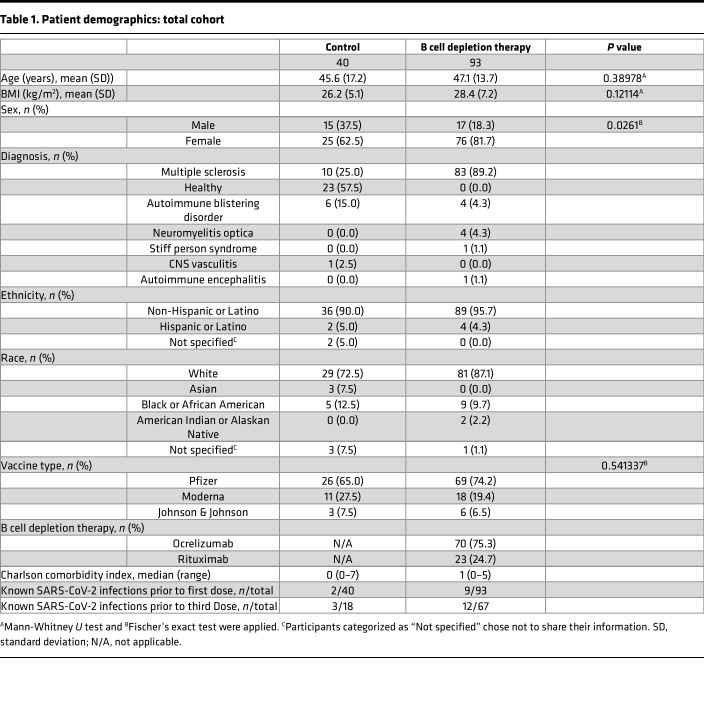
Patient demographics: total cohort

**Table 2:**
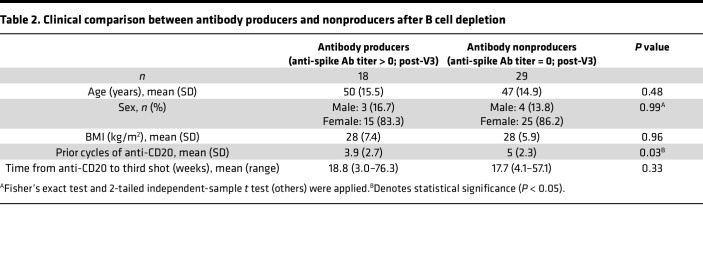
Clinical comparison between antibody producers and nonproducers after B cell depletion

**Table 3:**
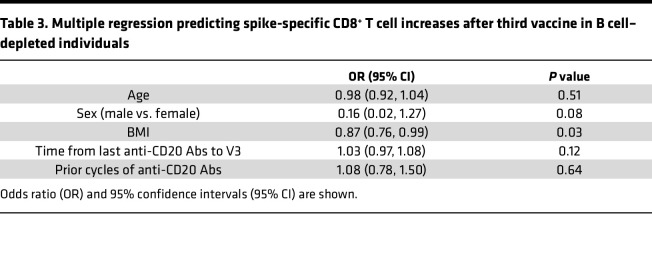
Multiple regression predicting spike-specific CD8^+^ T cell increases after third vaccine in B cell–depleted individuals

**Table 4:**
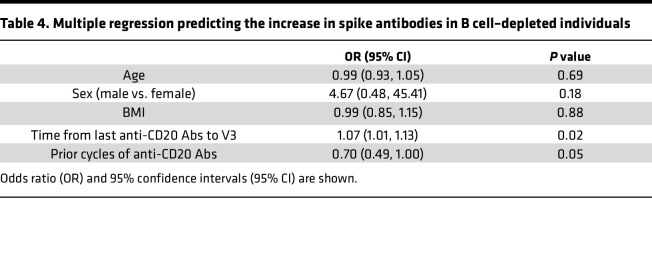
Multiple regression predicting the increase in spike antibodies in B cell–depleted individuals

## Version 1 08/22/2023

Electronic publication

## Version 2 09/01/2023

Funding information was added
