# Supplementary material for: Prior cycles of anti-CD20 antibodies affect antibody responses after repeated SARS-CoV-2 mRNA vaccination
Source: JCI Insight. 2023 Aug 22;8(16):e168102. doi: 10.1172/jci.insight.168102 (PMC10543713; doi:10.1172/jci.insight.168102)
Supplement: Supplemental data [file jciinsight-8-168102-s293.pdf]

Supple Fig. 1

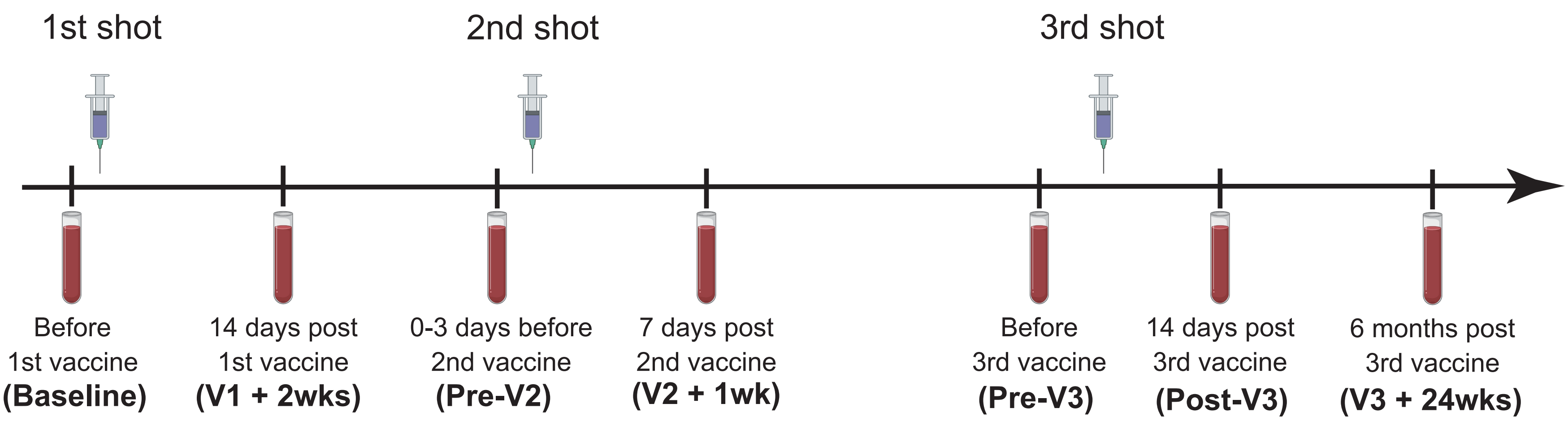

		(Baseline)	(V1 + 2wks)	(Pre-V2)	(V2 + 1wk)	(Pre-V3)	(Post-V3)	(V3 + 24wks)
Sera	**Total**	**75**	**76**	**71**	**64**	**85**	**82**	**39**
	**Ctrl**	**22**	**26**	**24**	**22**	**18**	**18**	**10**
	HD	12	14	13	11	9	9	6
	DC	10	12	11	11	9	9	4
	**BCDT**	**53**	**50**	**47**	**42**	**67**	**64**	**29**
PBMCs	**Total**	**40**	—	—	—	**58**	**58**	—
	**Ctrl**	**17**				**10**	**10**	
	HD	8	—	—	—	7	7	—
	DC	9				3	3	
	**BCDT**	**23**				**48**	**48**	

Supplemental Figure 1. Experimental study design
Longitudinal study design and time points collected pre- and post-SARS-CoV-2 mRNA vaccination for controls and patients with anti-CD20 mAbs. Total sample numbers at each timepoint were denoted and numbers in each group are shown. Sera were evaluated for antibodies and proteomics, and PBMCs were evaluated for spike-specific T cells and B cells. Ctrl; control subjects, BCDT; patients with B cell depletion therapy, HC; healthy controls, DC; disease controls

Supple Fig. 2

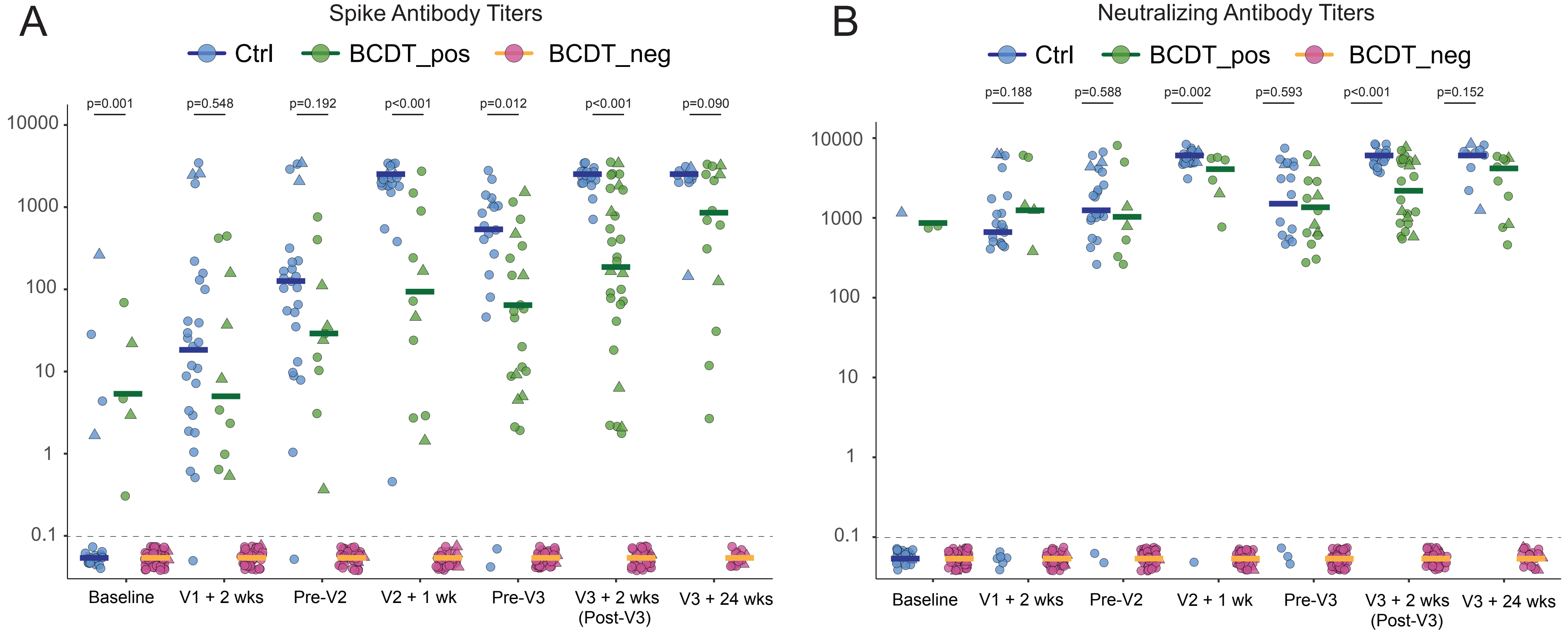

C

Multivariate logistic regression
for presence of anti-spike-antibodies among BCDT

	OR (95% CI)	p-value
Age	1.04 (1.00, 1.07)	0.07
Gender, M vs F	0.56 (0.16, 1.89)	0.35
BMI	1.04 (0.98, 1.11)	0.17
Time from last αCD20 Abs to V3	1.00 (0.98, 1.02)	0.79
Prior cycles of αCD20 Abs	0.82 (0.68, 0.97)	0.03
Previous COVID-19 infections, Yes vs No	4.96 (1.32, 18.56)	0.02

D

Multivariate logistic regression
for presence of anti-neutralizing-antibodies among BCDT

	OR (95% CI)	p-value
Age	1.03 (0.99, 1.07)	0.13
Gender, M vs F	0.53 (0.14, 2.05)	0.36
BMI	1.03 (0.97, 1.10)	0.37
Time from last αCD20 Abs to V3	1.00 (0.98, 1.03)	0.75
Prior cycles of αCD20 Abs	0.79 (0.64, 0.96)	0.02
Previous COVID-19 infections, Yes vs No	4.39 (1.24, 15.49)	0.02

Supplemental Figure 2. Humoral vaccine responses among patients with B cell depletion
A and B, Dot plots of anti-SARS-CoV-2 spike antibody titers (**A**) and neutralizing antibody titers (**B**) were evaluated from pre 1st vaccine (Baseline) to 6 months post 3rd vaccine (V3 + 24 weeks) among controls (Ctrl), SARS-CoV-2-seropositive patients with B cell depleted (B cell depleted_pos) and seronegative patients with B cell depleted (B cell depleted_neg). Circles represent participants without documented COVID-19 infections and triangles show those with a known infection at each time point. The median is marked by a horizontal line. The dotted line denotes the threshold of detection. Data were evaluated by two-tailed independent samples t-test. **C and D,** Multivariate logistic regression models predicting positive anti-spike antibody titers (**C**) or anti-neutralizing antibody titers (**D**) in B cell depleted subjects. Odds ratio (OR) and 95% confidence intervals (95% CI) are shown.

Supple Fig. 3

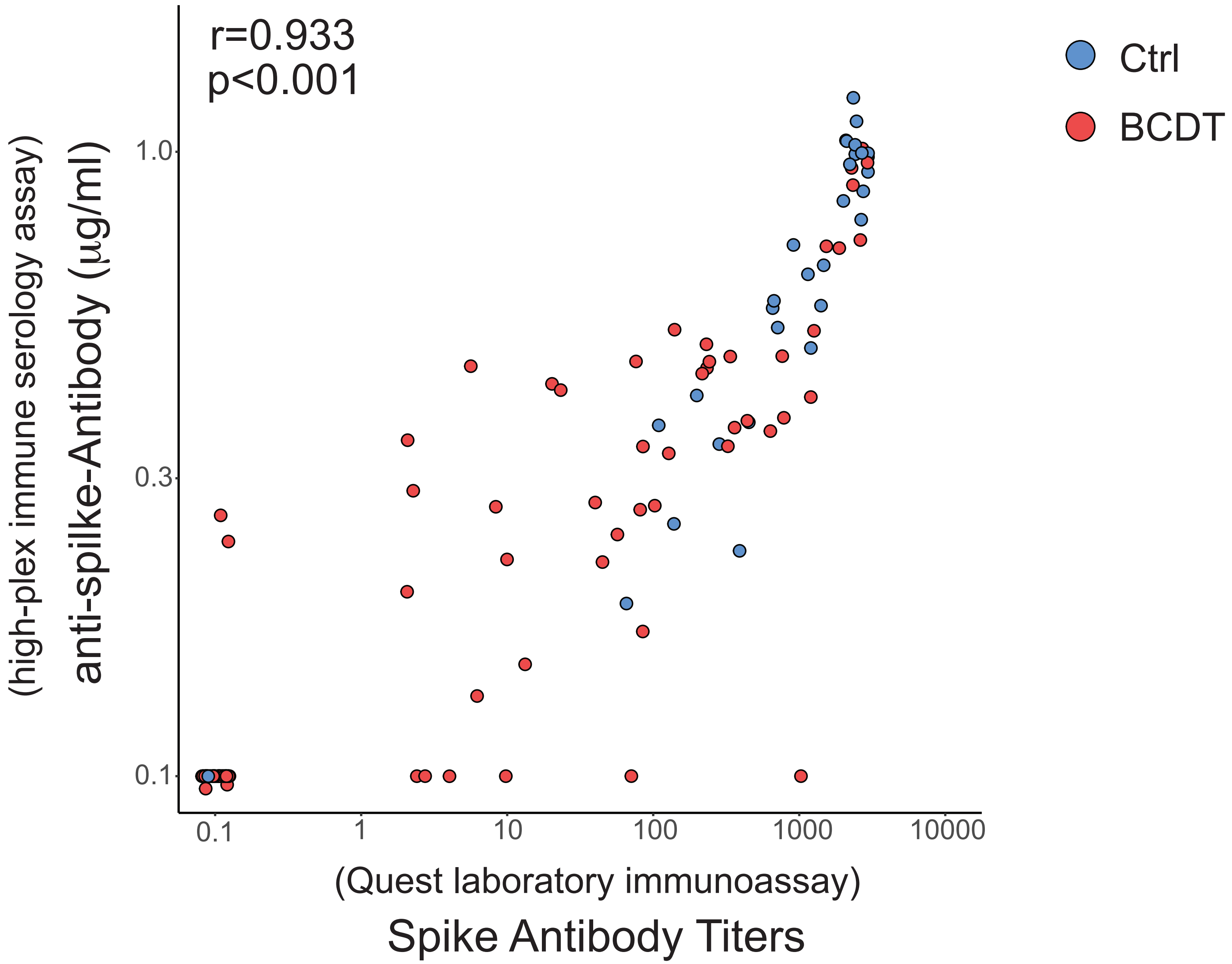

Supplemental Figure 3. Validation of our high-plex immune-serology assay
Correlation between anti-SARS-CoV-2 spike antibody titers from commercial immunoassays and the concentration (μg/ml) of anti-SARS-CoV-2 spike antibodies from our high-plex immune-serology assays. 156 samples (31 from controls and 125 from subjects with B cell depletion) were applied and correlation statistics were performed by two-tailed Spearman's rank correlation test.

Supple Fig. 4

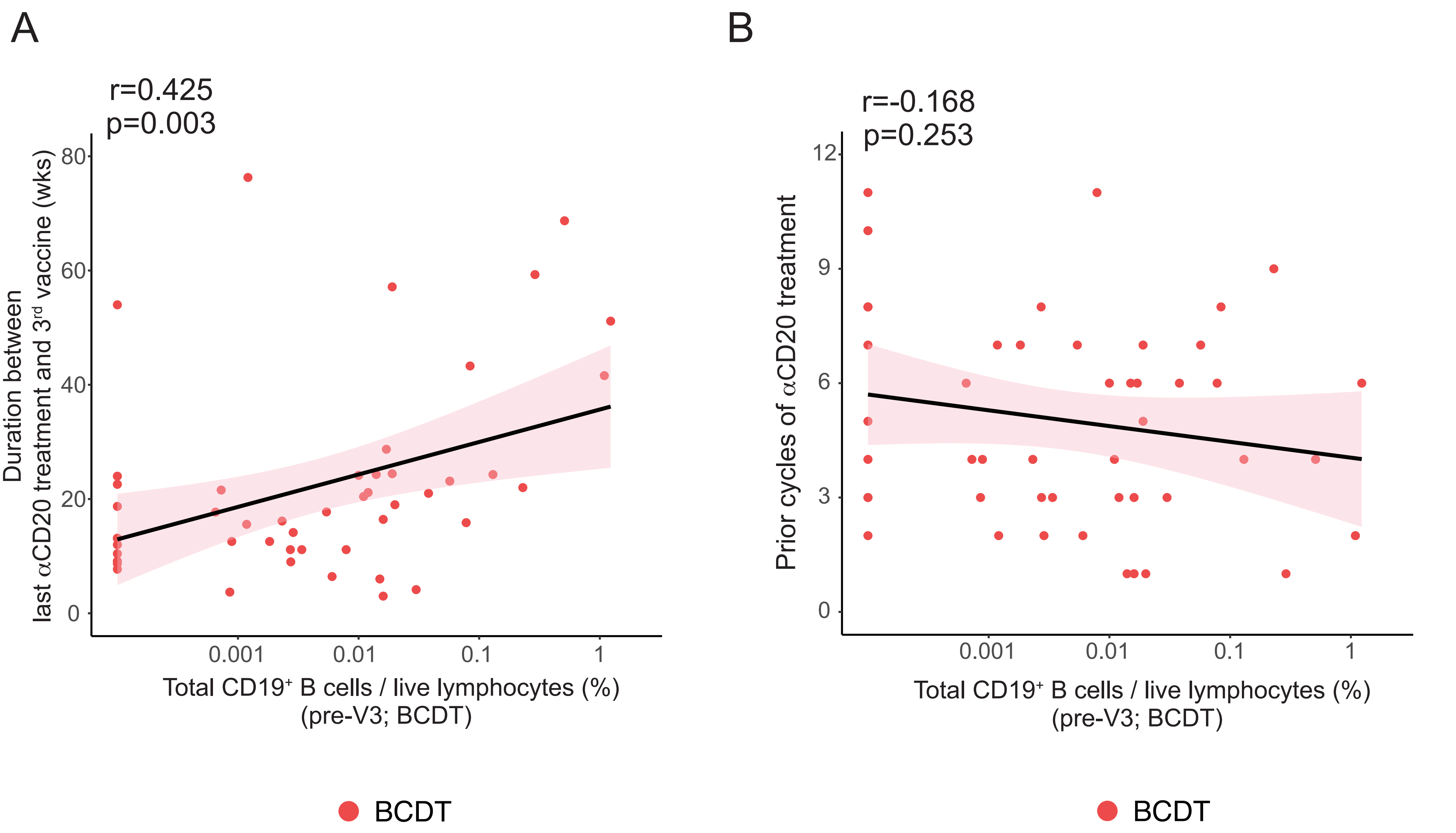

Supplemental Figure 4. Correlations between total CD19+ B cells in blood and clinical information
A and B, Correlation between total CD19+ B cells at pre-V3 and the duration between last cycles of anti-CD20 antibodies and 3rd vaccine (weeks) (**A**) or prior cycles of anti-CD20 antibodies at pre-V3 (**B**). Samples at pre-V3 of B cell depleted subjects (n=48) were applied. Linear regression is shown with 95% confidence interval (pink area) and correlation statistics by two-tailed Spearman's rank correlation test were performed.

Supple Fig. 5

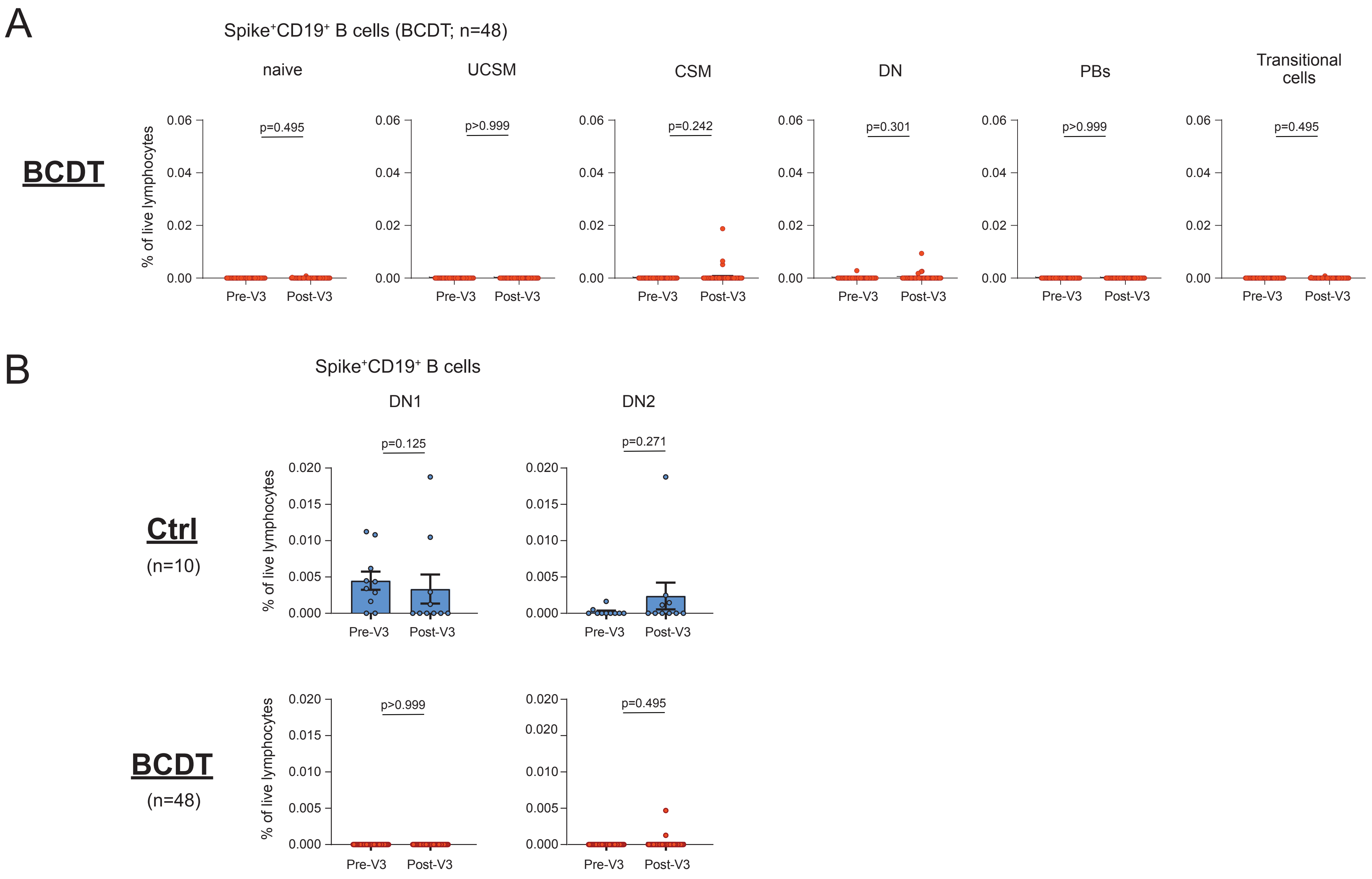

Supplemental Figure 5. Subsets of spike-specific B cells
A, The proportions of spike-specific B cells between pre-V3 and post-V3 in B cell depleted patients (n=48). UCSM; IgD+CD27+ unclass-switched memory B cells, CSM; IgD-CD27+ class-switched memory B cells, DN; IgD-CD27- double-negative B cells. **B** The proportions of DN cells between pre-V3 and post-V3. DN1; IgD-CD27-CD11c-CX-CR5+ double-negative 1 cells, DN2; IgD-CD27-CD11c+CXCR5- double-negative 2 cells.

Supple Fig. 6

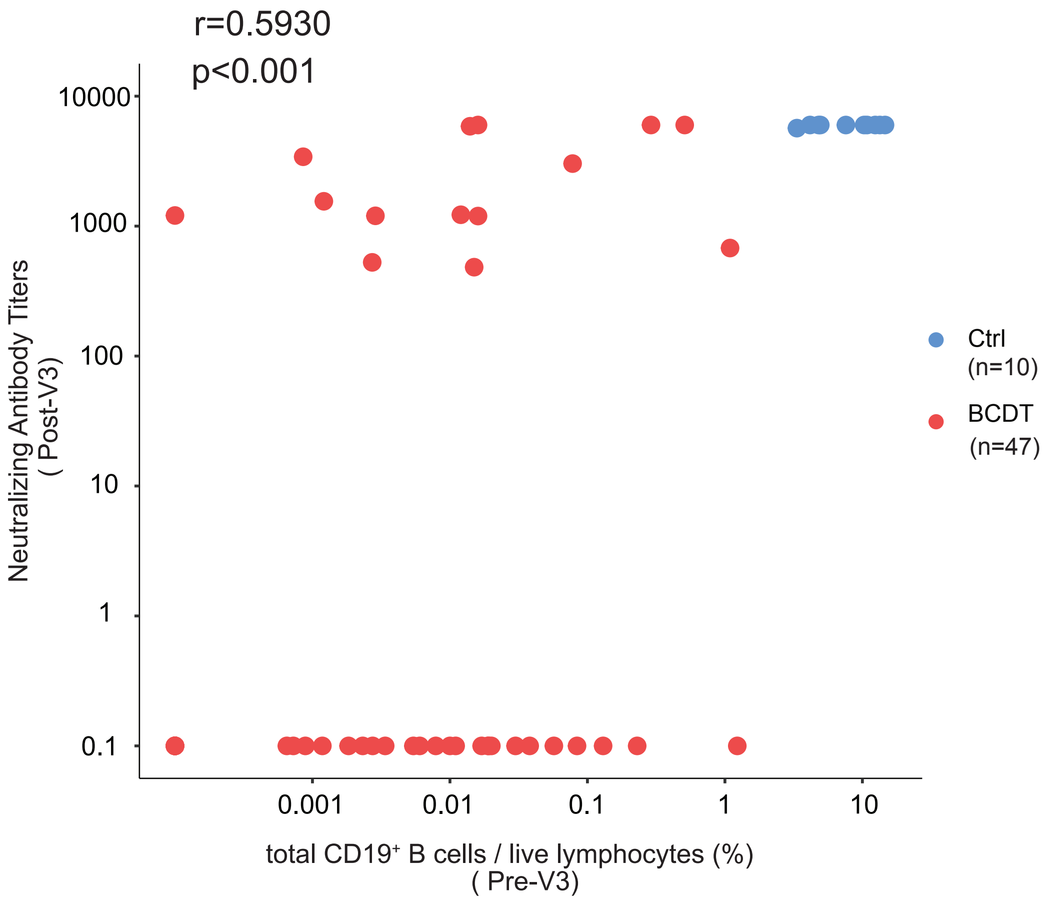

● BCDT (n=47)

	**Neut. Ab Titer > 0** (Post-V3)	**Neut. Ab Titer = 0** (Post-V3)	p-value
n	14	33	
Age (yrs), Mean (SD)	49 (15.7)	48 (15.0)	p=0.93
Sex, n(%)	M: 2 (14.3)<br>F: 12 (85.7)	M: 5 (15.2)<br>F: 28 (84.8)	p=1.00§
BMI (kg/m^2)<br>Mean (SD)	28.5 (7.6)	27.8 (6.0)	p=0.99
Prior Cycles of αCD20<br>Mean (SD)	3.6 (2.7)	5.4 (2.3)	p=0.01*
Time from αCD20 to 3rd shot (wks), Median (Range)	18.8 (3.0 - 76.3)	17.7 (4.1 - 57.1)	p=0.46

Supplemental Figure 6. Relationships between clinical characteristics and neutralizing antibodies
The proportion of CD19+ B cells at the pre-V3 timepoint was correlated with neutralizing antibody titers at the post-V3 timepoint in B cell depleted subjects (n=47). Correlation was performed using two-tailed Spearman's rank correlation test (left). Clinical information between neutralizing antibody producers (titers > 0 at post-V3; n=14) and antibody non-producers (titers = 0 at post-V3; n=33) after B cell depletion is shown (right). §denotes Fisher exact test; other p-values were calculated using two-tailed independent samples t-test. *denotes statistical significance (p<0.05).

Supple Fig. 7

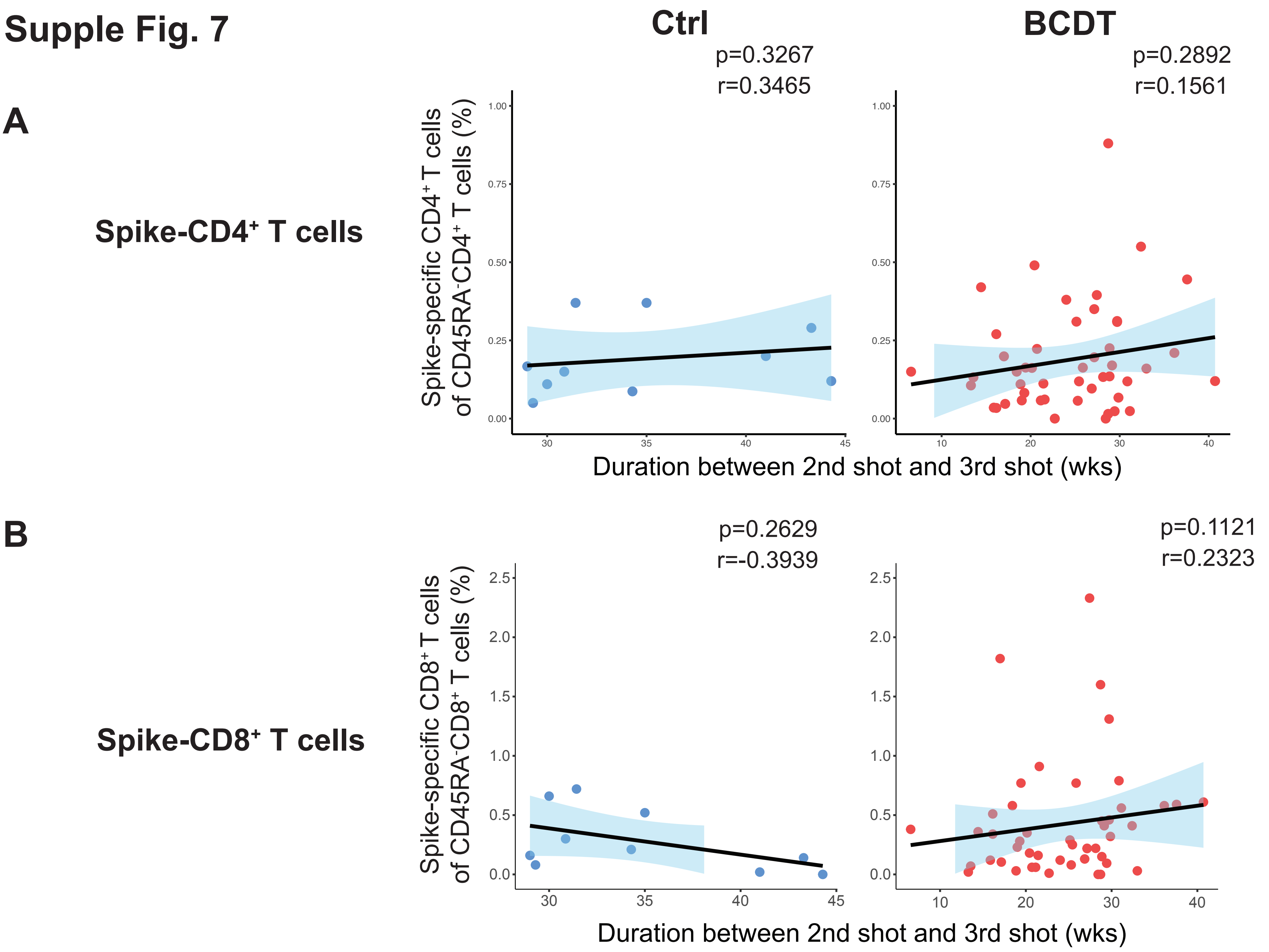

Supplemental Figure 7. Relationships between time to third vaccine and spike-specific T cell responses
A and B, The proportion of spike-specific CD4+ T cells (**A**) or CD8+ T cells (**B**) at the pre-V3 timepoint was correlated with the duration between 2nd and 3rd vaccines (Ctrl: n=10, BCDT: n=48). Linear regression is shown with 95% confidence interval (sky blue area). Correlation was performed using two-tailed Spearman's rank correlation test. Ctrl: control, BCDT: B cell depletion therapy.

Supple Fig. 8

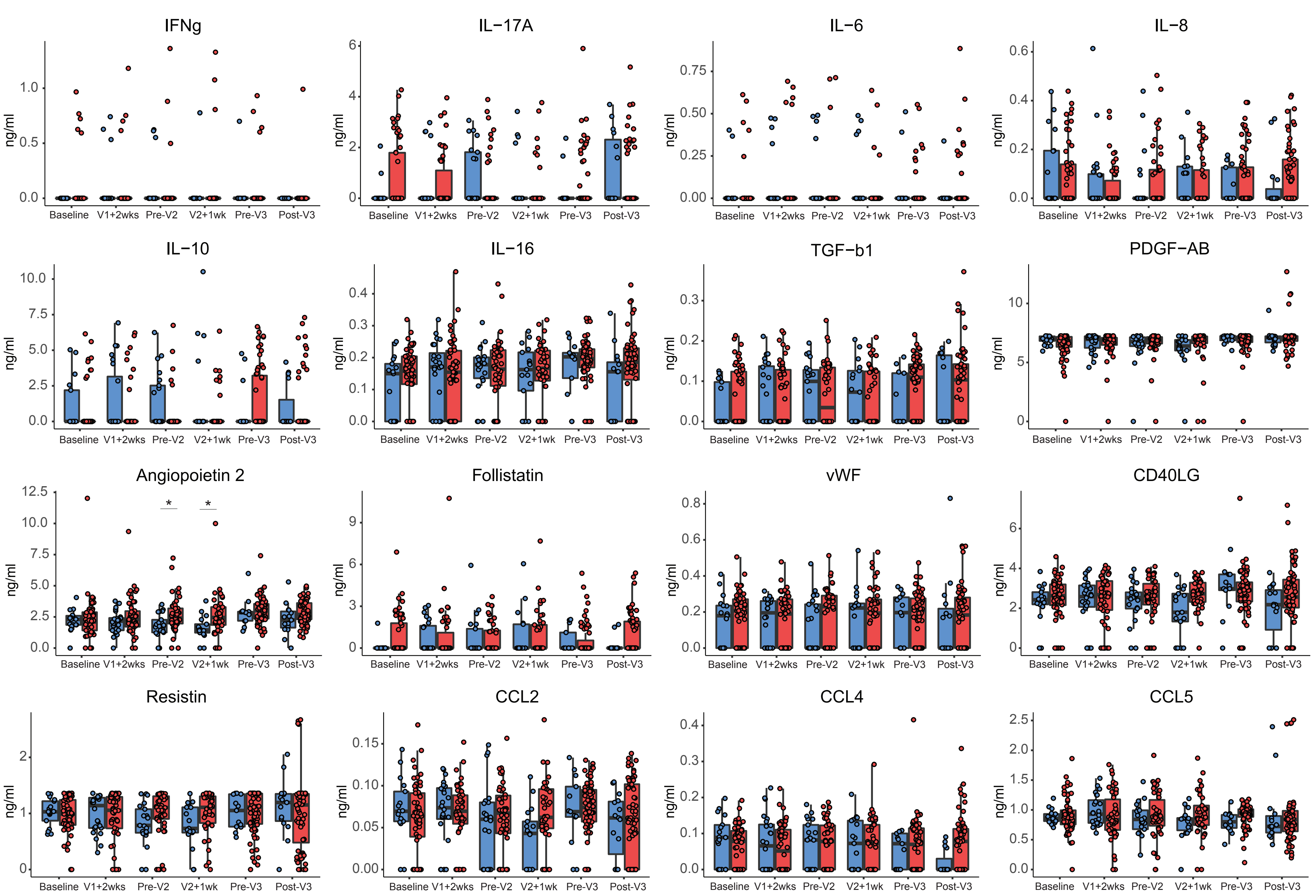

Supplemental Figure 8. Longitudinal proteomics data between controls and patients with B cell depletion therapy

The concentrations of each protein were evaluated cross-sectionally between controls and B cell depletion. A total of 432 samples were evaluated and numbers at each time point were as follows; Baseline (control; n=17, B cell depleted; n=57), V1+2 weeks (control; n=24, B cell depleted; n=50), Pre-V2 (control; n=21, B cell depleted; n=46), V2+1week (control; n=17, B cell depleted ; n=41), Pre-V3 (control; n=13, B cell depleted; n=67), Post-V3 (control; n=15, B cell depleted; n=64).

Supple Fig. 9

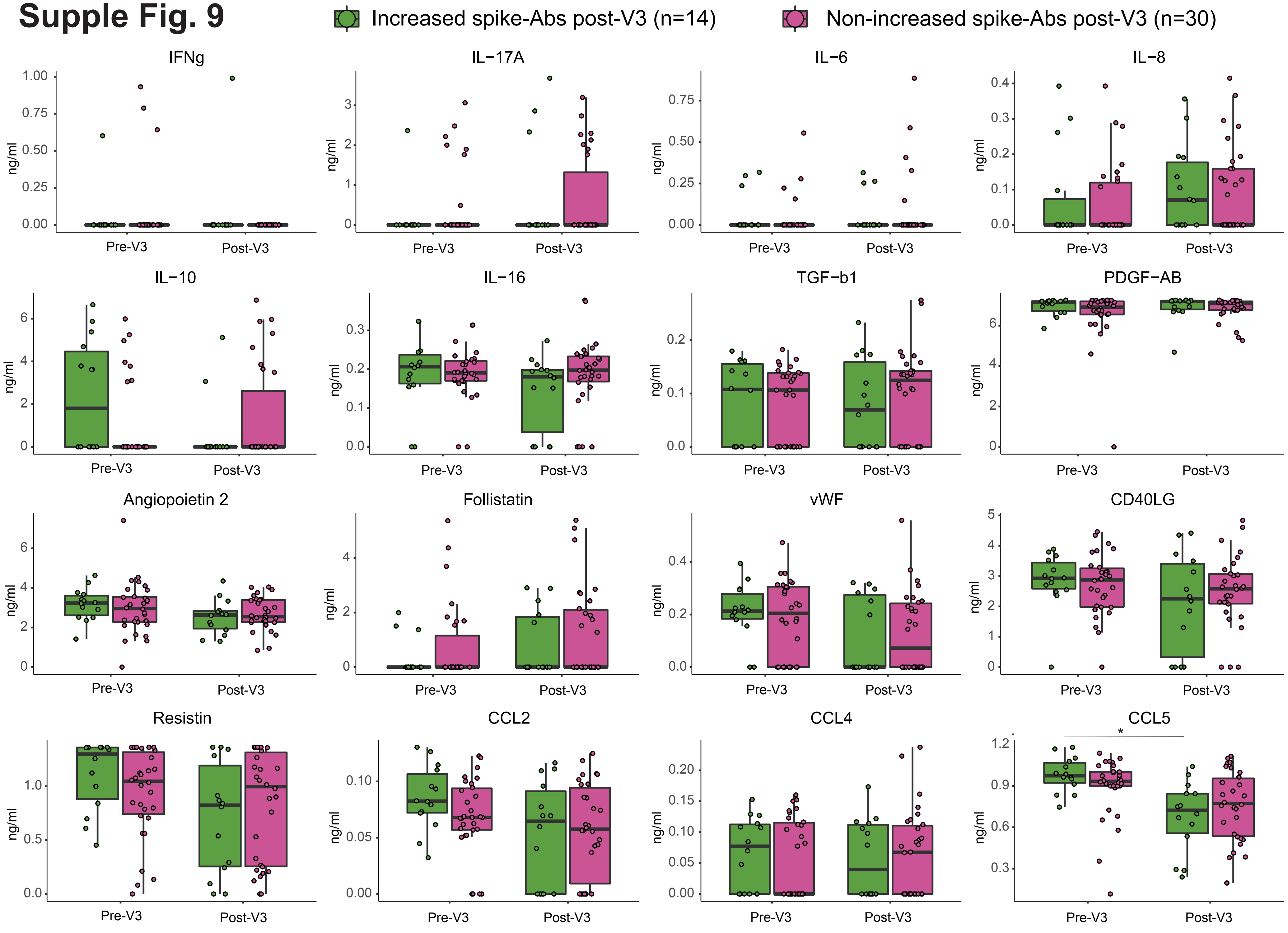

Supplemental Figure 9. The relationship between humoral immune responses and proteomics in B cell depletion
The concentrations of each protein pre- and post-V3 were evaluated between B cell depleted subjects with increased anti-spike antibody titers post-V3 (n=14) and subjects with no increase (n=30). Independent samples t-tests with Bonferroni correction were performed ($p<0.0125$ as significant).

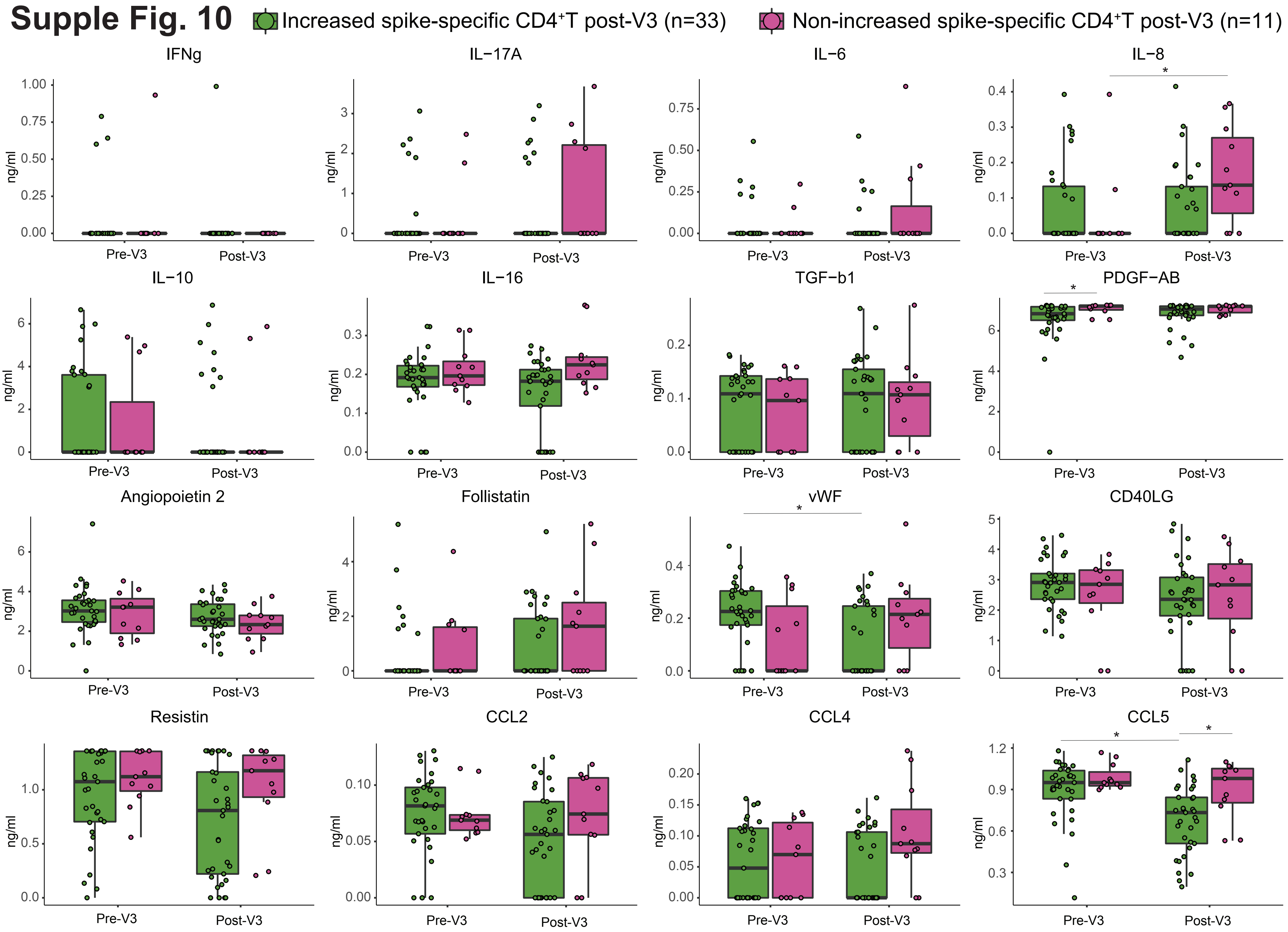

Supplemental Figure 10. The relationship between spike-specific CD4+ T cell responses and proteomics in B cell depleted patients
The concentrations of each protein pre- and post-V3 were evaluated between B cell depleted subjects with increased spike-specific CD4+ T cells post-V3 (n=33) and subjects with no increase (n=11). Independent samples t-tests with Bonferroni correction were performed ($p<0.0125$ as significant).

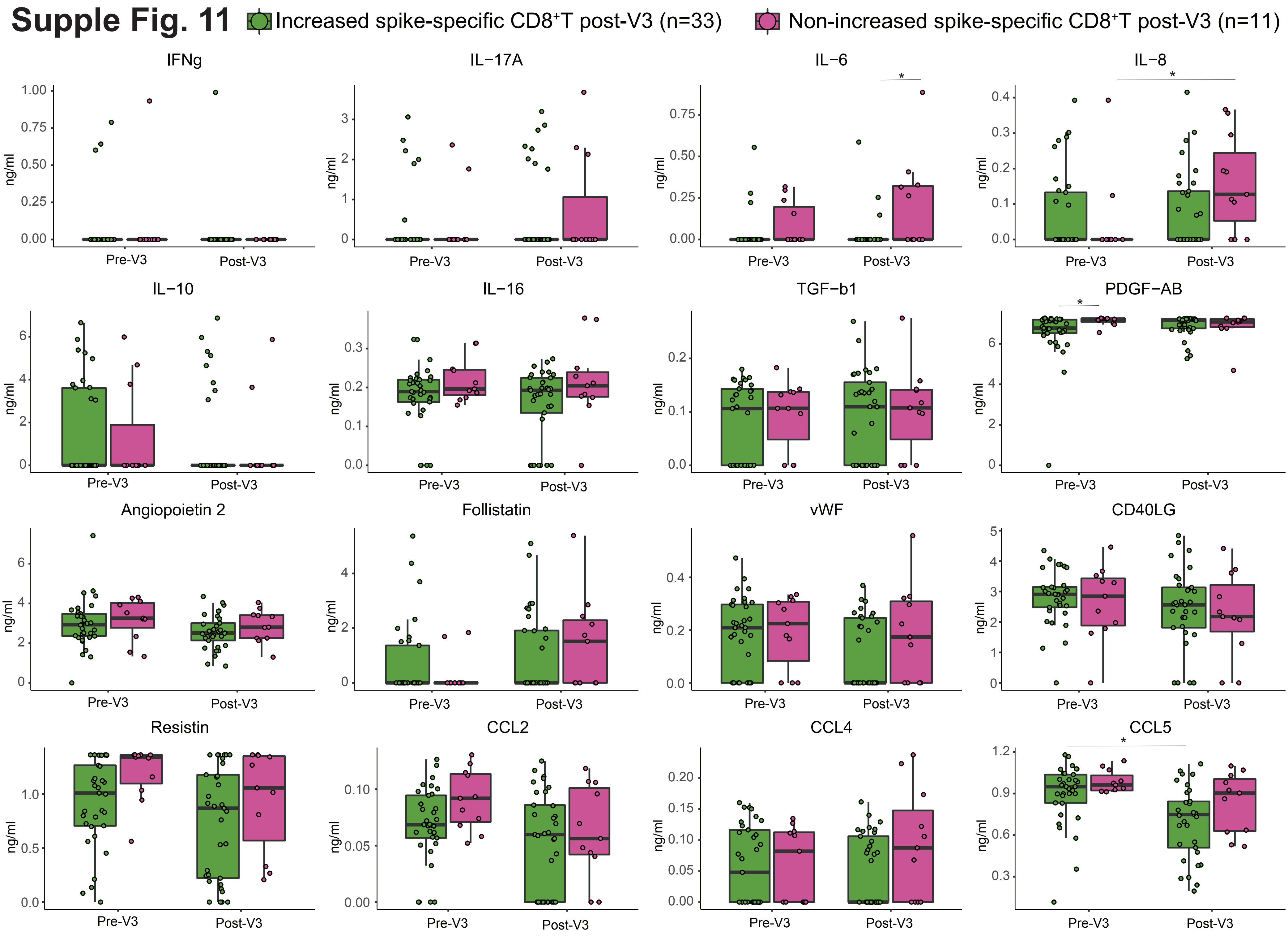

Supplemental Figure 11. The relationship between spike-specific CD8+ T cell responses and proteomics in B cell depleted patients
The concentrations of each protein pre- and post-V3 were evaluated between B cell depleted subjects with increased spike-specific CD8+ T cells post-V3 (n=33) and subjects with no increase (n=11). Independent samples t-tests with Bonferroni correction were performed ($p<0.0125$ as significant).

Supple Fig. 12

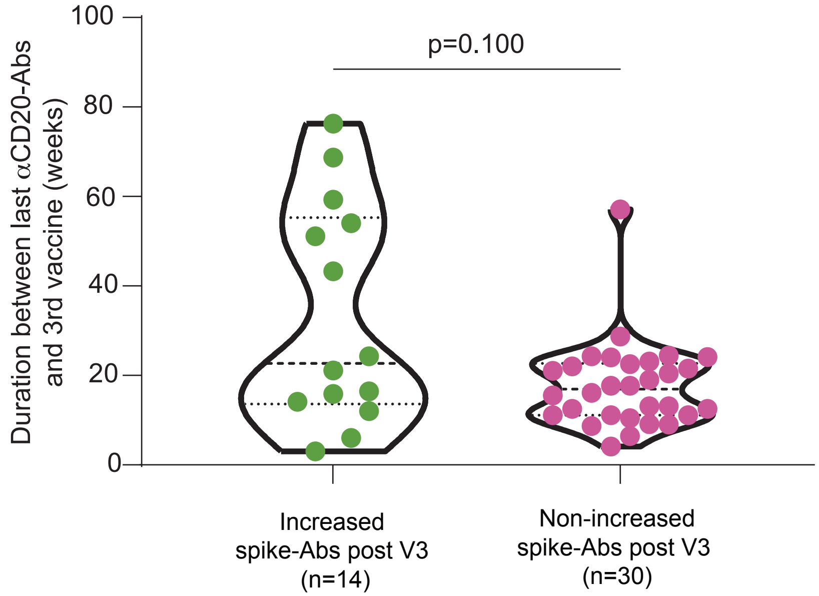

Supplemental Figure 12. The duration between last αCD20-Abs and 3rd vaccine in B cell depleted patients
Duration between last anti-CD20 antibodies and 3rd vaccine in B cell depleted subjects with increased anti-spike antibodies (n=14) and without an increase (n=30). Two-tailed independent samples t-test was performed.
